# Inter-phylum circulation of a beta-lactamase-encoding gene: a rare but observable event

**DOI:** 10.1128/aac.01459-23

**Published:** 2024-03-05

**Authors:** Rémi Gschwind, Marie Petitjean, Claudine Fournier, Julie Lao, Olivier Clermont, Patrice Nordmann, Alexander Mellmann, Erick Denamur, Laurent Poirel, Etienne Ruppé

**Affiliations:** 1 Université Paris Cité, INSERM, Université Sorbonne Paris Nord, IAME, Paris, France; 2 AP-HP, Hôpital Bichat, Laboratoire de Bactériologie, Paris, France; 3 Emerging Antibiotic Resistance, Medical and Molecular Microbiology, Faculty of Science and Medicine, University of Fribourg, Fribourg, Switzerland; 4 Swiss National Reference Center for Emerging Antibiotic Resistance, Fribourg, Switzerland; 5 INSERM European Unit (IAME, France), University of Fribourg, Fribourg, Switzerland; 6 University of Lausanne, University Hospital Center, Lausanne, Switzerland; 7 Institute of Hygiene, University Hospital Münster, Münster, Germany; 8 AP-HP, Hôpital Bichat, Laboratoire de Génétique Moléculaire, Paris, France; Columbia University Irving Medical Center, New York, USA

**Keywords:** antimicrobial resistance, beta-lactamase, horizontal gene transfer

## Abstract

Beta-lactamase-mediated degradation of beta-lactams is the most common mechanism of beta-lactam resistance in Gram-negative bacteria. Beta-lactamase-encoding genes can be transferred between closely related bacteria, but spontaneous inter-phylum transfers (between distantly related bacteria) have never been reported. Here, we describe an extended-spectrum beta-lactamase (ESBL)-encoding gene (*bla*_MUN-1_) shared between the Pseudomonadota and Bacteroidota phyla. An *Escherichia coli* strain was isolated from a patient in Münster (Germany). Its genome was sequenced. The ESBL-encoding gene (named *bla*_MUN-1_) was cloned, and the corresponding enzyme was characterized. The distribution of the gene among bacteria was investigated using the RefSeq Genomes database. The frequency and relative abundance of its closest homolog in the global microbial gene catalog (GMGC) were analyzed. The *E. coli* strain exhibited two distinct morphotypes. Each morphotype possessed two chromosomal copies of the *bla*_MUN-1_ gene, with one morphotype having two additional copies located on a phage-plasmid p0111. Each copy was located within a 7.6-kb genomic island associated with mobility. *bla*_MUN-1_ encoded for an extended-spectrum Ambler subclass A2 beta-lactamase with 43.0% amino acid identity to TLA-1. *bla*_MUN-1_ was found in species among the Bacteroidales order and in *Sutterella wadsworthensis* (Pseudomonadota). Its closest homolog in GMGC was detected frequently in human fecal samples. This is, to our knowledge, the first reported instance of inter-phylum transfer of an ESBL-encoding gene, between the Bacteroidota and Pseudomonadota phyla. Although the gene was frequently detected in the human gut, inter-phylum transfer was rare, indicating that inter-phylum barriers are effective in impeding the spread of ESBL-encoding genes, but not entirely impenetrable.

## INTRODUCTION

Beta-lactamases refer to enzymes catalyzing the hydrolysis of the beta-lactam ring, thereby inactivating the antibiotic properties of the molecule (1). While some Enterobacterales intrinsically harbor beta-lactamases, the biggest threat to health is due to the acquisition and exchange by pathogens of beta-lactamases-encoding genes, especially those encoding for extended-spectrum beta-lactamases (ESBLs) and carbapenemases. How the first move from the original host of the antibiotic resistance gene (ARG) and Enterobacterales species is barely known or based on *in silico* prediction in most instances (2, 3). Recently, Ebmeyer et al. described the origin of 37 ARGs found in Enterobacterales and provided evidence for the original gene-providing species for 27 groups of ARGs (2). Strikingly, 36/37 of transfer events occurred within the Pseudomonadota phylum (to which Enterobacterales belong). However, an exception was observed with *tet(X)*, which was proposed to originate from *Sphingomonas*, a genus from the Bacteroidota phylum (4). This observation supports that ARG transfers from other phyla to Pseudomonadota could spontaneously occur, albeit at a relatively rare frequency (3).

This lack of knowledge regarding ARG-providing species to Enterobacterales pointed at the intestinal microbiota as a potential reservoir (5). The dominant fraction of the intestinal microbiota is made of strict anaerobic bacteria which possess a vast diversity of ARGs including some encoding beta-lactamases (6), many of which have proven to be functional when transferred to *Escherichia coli* (7). However, ARGs from commensal anaerobic bacteria strongly differ from those found in Enterobacterales, stressing that their transfer to Enterobacterales would be particularly rare or would not persist so it would go unseen from the scientific community (8).

In a recent work, we searched for ARGs in 70,301 *E. coli* genomes from the EnteroBase using ARG databases including ARGs from intestinal strict anaerobic bacteria (9, 10). We could identify four ARGs presumably originating from non-Pseudomonadota, including a beta-lactamase-encoding gene also found in bacteria from the Bacteroidota phylum and that we propose to characterize in the present work.

## RESULTS

### Phenotypic characterization

**Fig 1:**
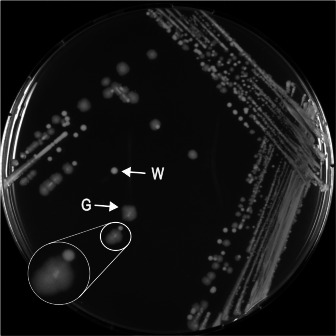
Morphological aspects of the two types of colonies (W: white colonies and G: gray colonies) observed after streaking the strain on lysogeny broth (LB) media.

An *E. coli* genome was identified as possessing a beta-lactamase-encoding gene which was only found in the ResFinderFG and Mustard databases (6, 9–11). The strain of interest belonged to the A phylogroup, sequence type 744/2 according to the Warwick University/Pasteur Institute schemes, respectively, and serotype Onovel132:H10, *fimH* allele 54. It was isolated in 2015 from a wound infection in a patient hospitalized at the University Hospital of Münster, Germany (12). The subcultures in LB media yielded two distinct morphotypes: white and regular shaped colonies or grayish and less regular colonies (Fig. 1). Both morphotypes were maintained in subsequent cultures.

From the antibiotic susceptibility testing, the *E. coli* strain isolate characterized by white and regular colonies displayed an ESBL phenotype with synergies being observed between clavulanic acid, cefotaxime, cefepime, and aztreonam (Fig. S1). Particularly, the strain showed a high level of resistance to cefuroxime, ceftazidime, aztreonam, and temocillin with MICs > 256 µg/mL (Table 1). It remained susceptible to carbapenems and cefoxitin, and to beta-lactam–beta-lactamase inhibitor combinations (clavulanic acid, tazobactam, and avibactam). Besides, the strain was resistant to cotrimoxazole and fluoroquinolones but was susceptible to aminoglycosides. The same phenotype was observed for gray colonies, except for some beta-lactam antibiotics (aztreonam, cefuroxime, cefotaxime, ceftazidime, and piperacillin) against which gray colonies were slightly less resistant (as observed from inhibition diameters).

**TABLE 1:** Minimal inhibitory concentrations of the white colonies, the *E. coli* TOP10 cloned or not cloned with the *bla*_MUN-1_ gene, and the kinetic parameters of purified MUN-1 beta-lactamase

Beta-lactam	MIC (µg/mL) for *Escherichia coli*			Kinetic measurement			
	Clinical strain	TOP10 (pBLA-x)	TOP10	*K*_cat_ (s^−1^)	*K*_*m*_ (µM)	*K*_cat_/*K*_*m*_ (µM^−1^ s^−1^)	*K*_*i*_ (µM)
Amoxicillin	>256	>256	4	ND^*a*^	ND	ND	ND
Amoxicillin + clavulanic acid	4	4	4	ND	ND	ND	ND
Ampicillin	ND	ND	ND	290	280	1	ND
Piperacillin	>256	>256	2	<0.01	ND	ND	0.0072
Piperacillin + tazobactam	4	4	2	ND	ND	ND	ND
Penicillin G	ND	ND	ND	210	95	2.2	ND
Temocillin	256	256	4	ND	ND	ND	ND
Ticarcillin	ND	ND	ND	<0.01	ND	ND	0.0036
Cephalothin	ND	ND	ND	30	15	2	ND
Cefuroxime	>256	>256	0.5	ND	ND	ND	ND
Ceftriaxone	16	16	0.12	ND	ND	ND	ND
Cefotaxime	4	2	0.12	<0.01	ND	ND	ND
Ceftazidime	>256	>256	0.25	<0.01	ND	ND	0.58
Ceftazidime + avibactam	0.06	0.03	0.25	ND	ND	ND	ND
Cefepime	4	2	0.06	<0.01	ND	ND	ND
Ceftolozane + tazobactam	0.06	0.06	0.06	ND	ND	ND	ND
Aztreonam	>256	>256	0.03	2	45	0.04	ND
Imipenem	0.25	0.25	0.25	<0.01	ND	ND	0.0013
Meropenem	0.03	0.03	0.03	<0.01	ND	ND	0.019
Ertapenem	0.03	0.03	0.03	<0.01	ND	ND	0.017

^*a*^ ND: not done.

### Beta-lactamase characterization

**Fig 2:**
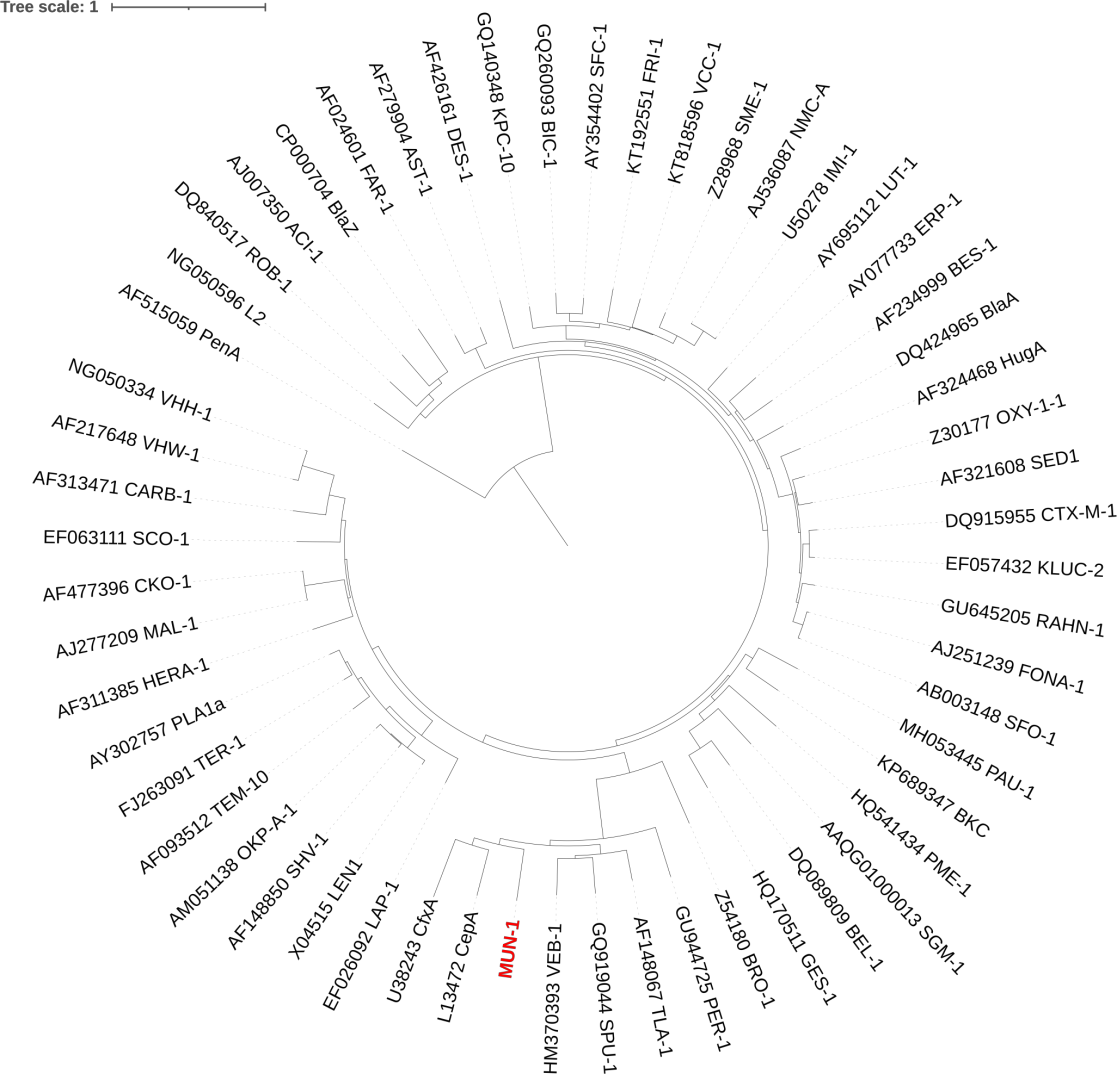
Phylogenetic tree of amino acid sequences of representative beta-lactamases found in the bacterial realm including the MUN-1 beta-lactamase (in red). Phylogenetic tree was rooted on PenA (found in the genus *Burkholderia*) which is distantly related from all the other beta-lactamases.

The MUN-1 amino-acid sequence was studied and compared to that of other beta-lactamases (Fig. 2) and was identified as an Ambler subclass A2 beta-lactamase (13). The closest beta-lactamases were TLA-1 (43.0% amino acid identity) and CepA (42.1% amino acid identity).

The cloned and expressed *bla*_MUN-1_ gene in *E. coli* TOP10 showed a similar resistance phenotype to the original strain (Table 1). The most potent inhibitor was clavulanic acid (50% inhibitory concentration; IC_50_ 0.32 nM), followed by tazobactam (IC_50_ 0.8 nM) and avibactam (IC_50_ 3.8 nM).

### Molecular characterization

White and gray *E. coli* strain isolates were sequenced using short-read and long-read technologies to identify the *bla*_MUN-1_ gene locations. The hybrid assembly produced two contigs for the gray colonies and three contigs for the white colonies (Table S1).

**Fig 3:**
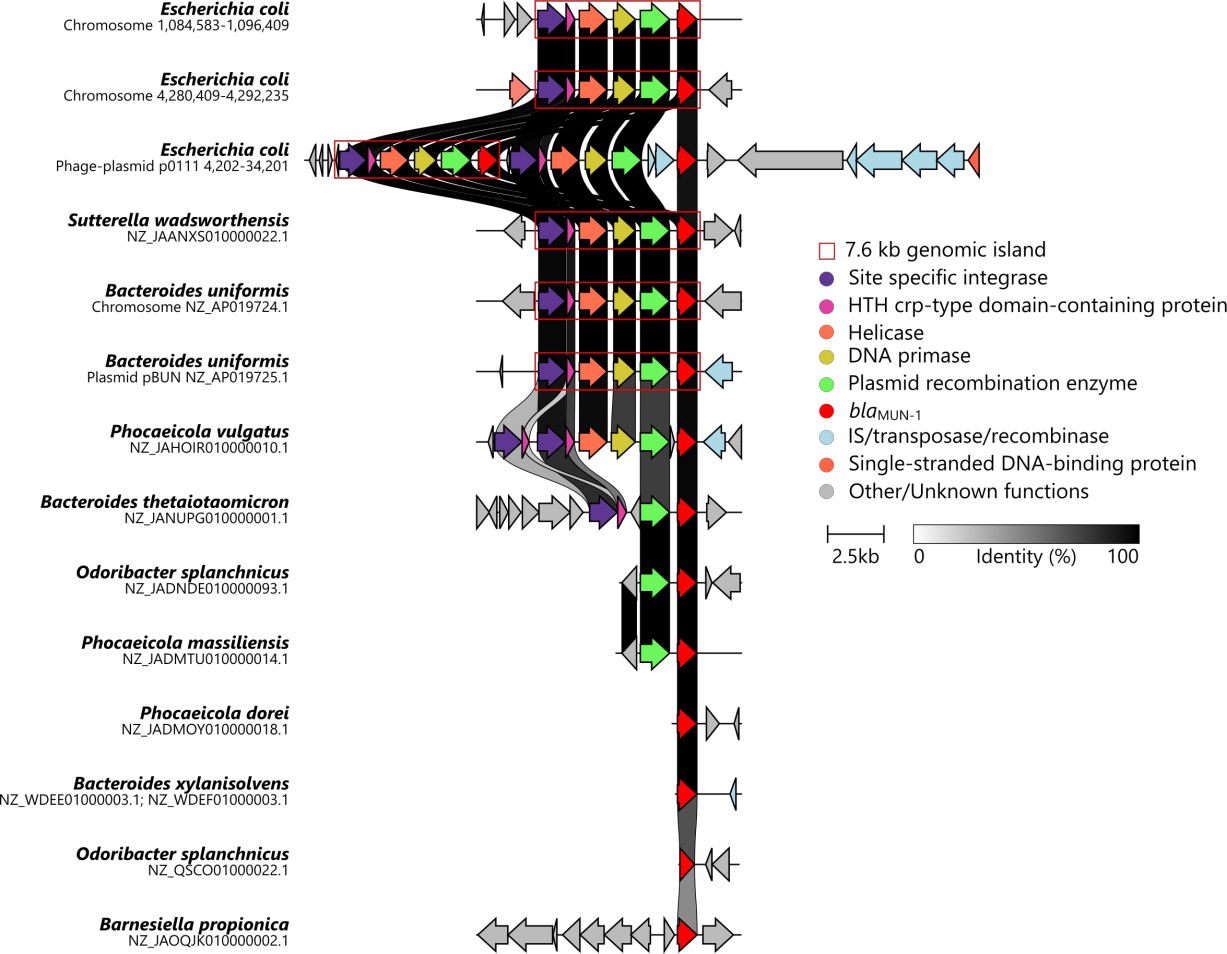
Genetic contexts showing the environment of the *bla*_MUN-1_ gene in different species. The first three lines describe the genetic contexts of each copy of the *bla*_MUN-1_ gene in the *E. coli* strain. Next, illustrative representatives from the RefSeq Genomes database were chosen for the following reasons: *Sutterella wadsworthensis* was the only other Pseudomonadota found to bear the *bla*_MUN-1_ gene; *Bacteroides uniformis* was the only genome in which copies of *bla*_MUN-1_ gene were found on a chromosome and on a plasmid; *Phocaeicola vulgatus*, *Bacteroides thetaiotaomicron*, *Odoribacter splanchnicus*, *Phocaeicola massiliensis*, *Phocaeicola dorei*, *Bacteroides xylanisolvens*, and *Barnesiella propionica* were chosen as they were the only genomes showing a genetic context that differed from the 7.6-kb genomic island. The red box delineates the 7.6-kb genomic island described in this work. The colors in the arrows correspond to the function of each gene. A nucleotide identity percentage between adjacent lines is displayed with a gray scale.

Circular bacterial chromosomes of 4,764,212 bp and 4,762,657 bp were identified for the gray and the white colonies, respectively. The ARG and virulence gene contents were similar in both morphotypes (Tables S2 and S3). Of note, 11 ARGs were located on a 26,418-bp antibiotic resistance genomic island (Fig. S2). The difference in the bacterial chromosome between the two morphotypes consisted in the presence of two additional insert sequences (0.78 kb each containing transposase-encoding genes) in the chromosome from the gray strain. One was cutting a glycosyltransferase-encoding gene and the other, an L,D-transpeptidase-encoding gene. *bla*_MUN-1_ was detected in two copies on the bacterial chromosome. Each *bla*_MUN-1_ copy was borne by a 7.6-kb genomic island (Fig. 3) annotated with five additional open reading frames encoding for a site-specific integrase, a helix-turn-helix crp-type domain-containing protein, a helicase, a DNA primase, and a plasmid recombination enzyme. The first 7.6-kb genomic island containing *bla*_MUN-1_ gene was located at 59.85 min and the second at 93.42 min on the *E. coli* genetic map (14). The GC content of the 7.6-kb genomic island was 45.4%, which was lower than the GC content of the entire chromosome (50.6%). The only shared characteristic found at the borders of each 7.6-kb genomic island was their low GC content, with an average of 34.3% GC in the 200-bp flanking each 7.6-kb genomic island copy.

A 127,245-bp circular p0111 phage plasmid bearing two copies of the *bla*_MUN-1_ gene was exclusively detected in the white colonies (Table S1). One copy was located on a 7.6-kb genomic island that was 100% identical to the ones found on the chromosome. A second 7.6-kb genomic island carrying the other gene copy was identified adjacent to the first 7.6-kb genomic island. However, this island was distinguished by the insertion of two insertion sequences (IS; IS3 family transposase ISEc52) between the *bla*_MUN-1_ gene and the plasmid recombination enzyme-encoding gene. Similar to the bacterial chromosome, the border of each 7.6-kb genomic island exhibited a lower GC content (mean of 31.5%) compared to the overall phage-plasmid GC content (46.6%). Next to the two 7.6-kb genomic islands, a 11-kb DNA fragment was shared between the p0111 and the chromosome suggesting recombination between the p0111 and the chromosome. Besides the bacterial chromosome, each morphotype had a circular IncFII plasmid of 60 kb. Of note, it did not embed any ARG.

### Distribution of the *bla*_MUN-1_ gene

**Fig 4:**
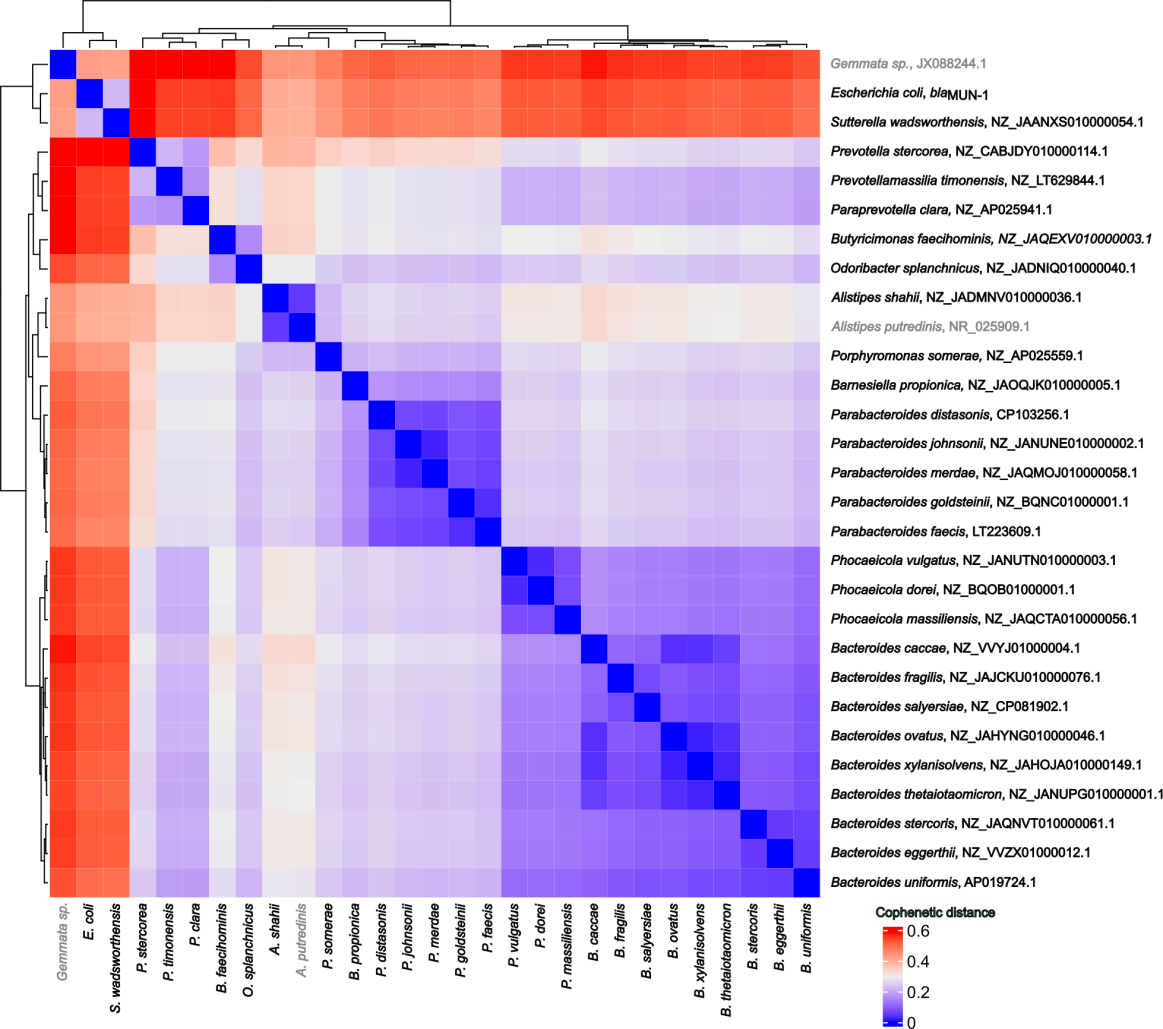
Cophenetic distance between species holding the *bla*_MUN-1_ gene based on the 16S rRNA-encoding gene. Heatmap represents the cophenetic distance between species. If no strain holding the *bla*_MUN-1_ gene from the species was found to hold a 16S rRNA-encoding gene, 16S rRNA-encoding gene was retrieved from strains that do not hold the *bla*_MUN-1_ gene (this was the case for *Alistipes putredinis* in gray). *Gemmata* sp. did not hold a *bla*_MUN-1_ gene in its genome but its 16S rRNA-encoding gene was used to root the phylogenetic tree.

**Fig 5:**
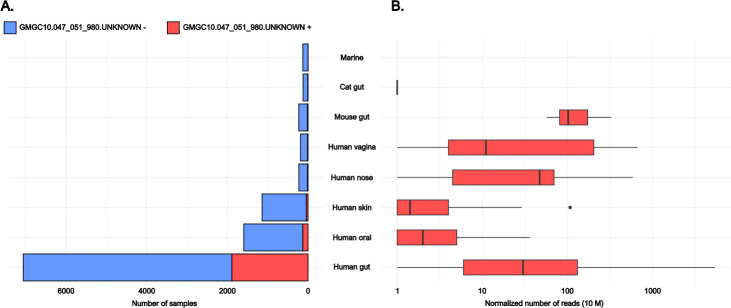
Frequency of the GMGC unigene GMGC10.047_051_980.UNKNOWN (100% identity and 96.4% cover in amino acid with the *bla*_MUN-1_ gene) in unigene sub-catalogs where it is found and the associated number of mapped reads. (A) Number of samples found in each sub-catalog containing (in red) or not (in blue) the GMGC unigene GMGC10.047_051_980.UNKNOWN. (B) Boxplot representation of normalized number of reads (out of 10 million reads) mapping onto the GMGC unigene GMGC10.047_051_980.UNKNOWN in each GMGC sub-catalog where it was found. The normalization takes into account the size of the gene and the number of reads in each sample from the sub-catalogs. First, median, and third quartiles are represented in each box. Whiskers extend from the hinge to the smallest/largest value at most/no further than 1.5× inter-quartile range from the hinge. Points represent outliers.

We searched for *bla*_MUN-1_ using RefSeq Genomes databases from NCBI and BLASTN (70% nucleotide identity, 80% coverage) (15). A total of 125 hits were obtained, with the *bla*_MUN-1_ gene being present in 28 species (100% nucleotide identity and coverage), 27 of which belonged to the Bacteroidota phylum, specifically within the Bacteroidales order (Table S4). A unique hit was detected in the Pseudomonadota phylum with *Sutterella wadsworthensis*. Similar to our *E. coli* strain, the *bla*_MUN-1_ gene was sporadically detected in multiple copies (maximum of six copies/genome in *Bacteroides uniformis*). We could not determine whether the sequence holding the *bla*_MUN-1_ gene was chromosomal or plasmidic, one exception being a *B. uniformis* genome (AP019724.1 and AP019725.1) bearing two copies of the 7.6-kb genomic island (containing the *bla*_MUN-1_ gene), one being on a plasmid surrounded by sequences annotated as IS256 family transposase and site-specific integrase. Interestingly, two sequences on this plasmid were annotated as phage protein, yet none had homologs located on the p0111 phage plasmid found in the *E. coli* strain. Some variants of the *bla*_MUN-1_ gene were found in *Bacteroides salyersiae*, *Bacteroides xylanisolvens*, *Parabacteroides distasonis*, *Leyella stercorea*, and *Phocaeicola vulgatus* (97.0%–99.9% nucleotide identity). Additionally, *Barnesiella propionica* was shown to bear a gene with 71.9% nucleotide identity and 88% coverage to *bla*_MUN-1_ gene. Of note, *bla*_MUN-1_ was not constantly found in any species (Table S4). Using a phylogenetic tree based on the 16S rRNA-encoding gene sequences of each species found to possess *bla*_MUN-1_, we observed the closest species to *E. coli* were *S. wadsworthensis* (cophenetic distance 0.22) and two species from the *Alistipes* genus (cophenetic distance 0.40; Fig. 4). The most distant species bearing *bla*_MUN-1_ was *L. stercorea* (cophenetic distance 0.61). Besides, MGnify and the global microbial gene catalog (GMGC) databases were used to analyze the distribution of the *bla*_MUN-1_ gene in various environments (16, 17). *bla*_MUN-1_ was also detected mostly in bacteria from the Bacteroidales order (86%–100% of the hits; Tables S5 and S6). We identified a close homolog to *bla*_MUN-1_ in the GMGC (GMGC10.047_051_980.UNKNOWN—*Prevotellamassilia timonensis*—100% amino acid identity and 92.4% cover). It was detected in several sub-catalogs but mainly in the human gut sub-catalog where it was found in 26.8% of the samples, with a mean relative abundance of 104.5/10 M reads (median: 12, min: 0, max: 5,371; Fig. 5).

*bla*_MUN-1_ was in most instances borne by the same 7.6-kb genomic island, also found in the *E. coli* strain except for 9 out of 125 hits with distinct genetic contexts (Fig. 3). In a *P. vulgatus* strain (NZ_JAHOIR010000010.1), *bla*_MUN-1_ was held by a 7.6-kb genomic island with 82.0% nucleotide identity. Then, in a *Bacteroides thetaiotaomicron* strain (NZ_JANUPG010000001.1), it was located on a shorter version of the 7.6-kb genomic island (63% cover) which consisted of the *bla*_MUN-1_ gene (100% cover, 100% identity), the plasmid recombination enzyme (100% cover, 99.9% identity), and the site-specific integrase-encoding genes (99% cover, 80.60% identity). In six cases, the *bla*_MUN-1_ gene was found at the edges of contigs, making it challenging to confirm the presence of the complete 7.6-kb genomic island (Fig. 3). Finally, a distinct genetic context was identified for the variant of *bla*_MUN-1_ (71.9% nucleotide identity) detected in *B. propionica*.

## DISCUSSION

The detection of the *bla*_MUN-1_ gene, encoding an ESBL, in both Bacteroidota and Pseudomonadota phyla, suggests the possibility of inter-phylum transfer of ESBL-encoding genes.

The characterized MUN-1 beta-lactamase was an Ambler subclass A2 beta-lactamase with an ESBL phenotype (13). Notably, it conferred resistance to several beta-lactam antibiotics, including temocillin, which is unusual among class A beta-lactamases (18). While it showed high MICs for piperacillin or ceftazidime, no hydrolysis of the compounds was detected. This could be due to the strong binding of the enzyme to the substrate (acylation step) but without the final step of deacylation that would lead to hydrolysis of the beta-lactam. Therefore, the substrate is not able to act as an antibiotic due to this strong binding but no hydrolysis rate can be detected from the method we used (19).

The distribution analysis revealed that *bla*_MUN-1_ was predominantly present in bacteria belonging to the Bacteroidales order with a single exception in a *S. wadsworthensis* genome. This suggests that inter-phylum transfer of the *bla*_MUN-1_ gene has indeed occurred at least once. The *bla*_MUN-1_ gene was most commonly found in association with the conserved 7.6-kb genomic island. The GC content of this genomic island was closer to that of *Bacteroides* than *E. coli*, suggesting a relatively recent inter-phylum transfer event. The distribution analysis of the gene homologous to the *bla*_MUN-1_ gene in the GMGC catalog revealed that it was predominantly detected in the human gut sub-catalog, in more than a quarter of the human gut samples. This supports that the transfer of *bla*_MUN-1_ occurred between *E. coli* and intestinal bacteria, either in the gut or in situations such as wounds.

Contamination of sequencing data by beta-lactamase-encoding genes associated with Taq polymerase producers can occur (20, 21). Here, several copies of *bla*_MUN-1_ were detected at several locations of the chromosome and the p0111 phage plasmid, both circular, suggesting that it does not come from contamination. Moreover, the beta-lactamase-encoding gene usually found as a contaminant in sequencing data is usually *bla*_TEM-1_, which is found here in the circular chromosome in the resistance genomic island with other ARGs. Yet, the beta-lactamase-encoding gene we extensively describe, *bla*_MUN-1_, encodes for MUN-1 whose closest homologs are not from the TEM family but TLA-1 and CepA which are found in *E. coli* and *Bacteroides* genus, respectively (22–24).

This paper has limitations. First, we could not determine the precise progenitor of *bla*_MUN-1_ because of its association with mobility. Moreover, no species constantly carrying *bla*_MUN-1_ could be identified. The precise genetic events leading to the presence of *bla*_MUN-1_ also remain hypothetical. The *E. coli* strain exhibited two morphotypes, one of which harbored an additional p0111 phage plasmid carrying two extra copies of the *bla*_MUN-1_ gene. These repetitive regions and mixed strains complicate the sequencing data analysis but the combination of short-read and long-read sequencing technologies undoubtedly facilitated read assembly and allowed the identification of this transfer between the chromosome and p0111. Yet, it cannot definitively establish the involvement of p0111 in horizontal gene transfer (HGT). The P1 phage-plasmid subgroup, of which p0111 is a member, is specifically found in *E. coli*. It has been associated with ARGs but was not found in Bacteroidota phyla so far (25, 26). The *bla*_MUN-1_ gene was also detected on a plasmid in *B. uniformis*, raising the possibility that this plasmid contributed to the inter-phylum transfer event. *In vitro* experiments demonstrated that the transformation of *E. coli* with a plasmid from *Bacteroides fragilis* was possible but conjugation between these two species was unsuccessful (27). However, *in vitro* experiments between two strains do not reflect a complex bacterial ecosystem. Inter-phylum transfer of DNA, including conjugation between Bacteroidota and Pseudomonadota, was shown to be possible within complex bacterial communities (8, 28, 29). *A. putredinis* and *S. wadsworthensis* are the closest related Bacteroidota and Pseudomonadota species based on their 16S rRNA-encoding genes but we cannot state which bacteria were involved in this HGT. However, the 7.6-kb genomic island should be involved as it is found in both phyla and is composed of genes associated with recombination events. The gene annotated as a plasmid recombination enzyme-encoding gene using Bakta was annotated as a mobilization protein-encoding gene in NCBI. This gene is linked to the relaxase domain of MobM and is responsible for recombination in a site-specific manner. The *E. coli* strain from this study could have acquired DNA from a Bacteroidales species or a *S. wadsworthensis* harboring *bla*_MUN-1_, with subsequent transpositions of the 7.6-kb element on p0111 and the chromosome.

Here was the first, to our knowledge, evidence of a shared ESBL-encoding gene between Bacteroidota and Pseudomonadota phyla. This observation shows that ESBL-encoding gene transfers between distantly related species can spontaneously occur. How such transfer actually occurred and why it has not widely spread subsequently remain to be answered.

## MATERIALS AND METHODS

### Bioinformatic analyses

In our previous work (9), we identified a putative beta-lactamase-encoding gene sharing 100% nucleotide identity with a beta-lactamase-encoding gene from ResFinderFG (beta_lactamase|KU546399.1|feces|AMX, 100% identity, 93.9% cover) and Mustard (MC3.MG60.AS1.GP1.C4251.G1). We propose the name *bla*_MUN-1_ with regard to the city where the original strain was collected (Münster, Germany).

### Strain characterization

The *E. coli* strain with the beta-lactamase-encoding gene was re-tested for antibiotic susceptibility by the disk diffusion method on Mueller-Hinton agar according to the CASFM/EUCAST guidelines (2022 v1.0) and re-sequenced using Illumina (San Diego, CA, USA) and Oxford Nanopore (Oxford Nanopore Technologies, UK) chemistries (Flongle R9.4.1). The quality of Illumina and Nanopore reads was assessed using FastQC (v0.11.9). Trim galore (v0.6.7) was used to remove Illumina adapters and trimmed reads with a quality threshold of 30. The hybrid assembly of Illumina and Nanopore reads was performed using Unicycler (v0.4.9b) (30). The phylogroup of the strain was performed using the ClermonTyping (v23.06.05) tool and the sequence type with MLST (v2.19.0). Serotype and virulence genes were characterized using the ABRicate (v1.0.0) software and the ecoh and a home-made database, respectively. The *fimH* allele was determined using FimTyper (v1.1). ARGs were identified using the Diamond software (v2.1.8) and the ResFinder database (v4.0) (31). PlasmidFinder was used to characterize plasmid incompatibility groups. Contigs were annotated using Bakta (v1.8.2) (32, 33).

### Distribution of the *bla*_MUN-1_ gene

The distribution of *bla*_MUN-1_ and potential variants was assessed using BLASTN (70% identity, 80% coverage) online with RefSeq Genomes database from NCBI (as of 24 August 2023) (15). Its genetic environment was annotated using Bakta (v1.8.2) and visualized using Clinker (34). Cophenetic distance between each species bearing the *bla*_MUN-1_ gene was determined using their 16S rRNA-encoding gene. If no 16S rRNA-encoding gene could be found in any representative species bearing the *bla*_MUN-1_ gene, a 16S rRNA-encoding gene sequence from a non-bearing species was used. The 16S rRNA-encoding genes were used for alignment with MAFFT (v7.407), and a phylogenetic tree was made using IQ-TREE (v1.6.9, with ultrafast bootstrap and general time reversible model) (35–37). Additionally, *bla*_MUN-1_ was also searched in the GMGC and in MGnify (16, 17). The distribution, relative abundance, and frequency of the best hit obtained with GMGC were also analyzed in the catalog.

### Characterization of MUN-1

The *bla*_MUN-1_ gene was translated into protein and aligned with other beta-lactamases retrieved from the ResFinder (v4.0) database using MAFFT (v7.407). To assess the phylogenetic distance between each beta-lactamases, a phylogenetic tree was made using IQ-TREE (v1.6.9, with ultrafast bootstrap and LG model). The *bla*_MUN-1_ gene was cloned into a pTOPO-kanR vector using the pCR-Blunt TOPO cloning kit (Invitrogen) using specific primers spanning the full gene in order to express the whole protein. The resulting recombinant plasmid was transformed by heat shock into *E. coli* TOP10 (pTOPO/*bla*_MUN-1_).

Purification of the MUN-1 beta-lactamase was carried out by ion-exchange chromatography, and its molecular mass was determined by SDS-12% PAGE (GeneScript) analysis. Purified beta-lactamase was used for kinetic measurements. IC_50_ values were determined for clavulanic acid, tazobactam, and avibactam (detailed protocol in the Supplementary Materials).

## Data Availability

Illumina and Nanopore reads were deposed under the BioProject PRJNA694822. Assemblies and gene sequences described here can be found at: https://doi.org/10.5281/zenodo.10560075. *bla*_MUN-1_ gene was deposited on NCBI under the accession number PP229523PP229523.

## SUPPLEMENTAL MATERIAL

Fig. S1 aac.01459-23-s0001.TIF Antibiotic susceptibility testing.

Fig. S2 aac.01459-23-s0002.TIF Antibiotic resistance genomic island found on the chromosome of the *E. coli* morphotypes.

Additional experimental details aac.01459-23-s0003.pdf Additional details about beta-lactamase characterization.

Table S1 aac.01459-23-s0004.pdf Summary of assemblies obtained using Unicycler for white and gray *E. coli* morphotypes.

Table S2 aac.01459-23-s0005.pdf Antibiotic resistance genes (ARGs) found in the chromosome of both *E. coli* morphotypes using ResFinder (v4.0).

Table S3 aac.01459-23-s0006.pdf ABRicate analysis of the virulence gene found in the genome of the *E. coli* morphotypes.

Table S4 aac.01459-23-s0007.pdf Hits obtained analyzing the blaMUN-1 gene distribution using BLASTN against RefSeq genomes database.

Table S5 aac.01459-23-s0008.pdf Hits obtained analyzing the blaMUN-1 gene distribution using MGnify.

Table S6 aac.01459-23-s0009.pdf Hits obtained analyzing the MUN-1 protein distribution using the GMGC catalog.

ASM does not own the copyrights to Supplemental Material that may be linked to, or accessed through, an article. The authors have granted ASM a non-exclusive, world-wide license to publish the Supplemental Material files. Please contact the corresponding author directly for reuse.
