# Supplementary figures and images for: Inter-phylum circulation of a beta-lactamase-encoding gene: a rare but observable event
Source: Antimicrob Agents Chemother. 2024 Mar 5;68(4):e01459-23. doi: 10.1128/aac.01459-23 (PMC10989005; doi:10.1128/aac.01459-23)

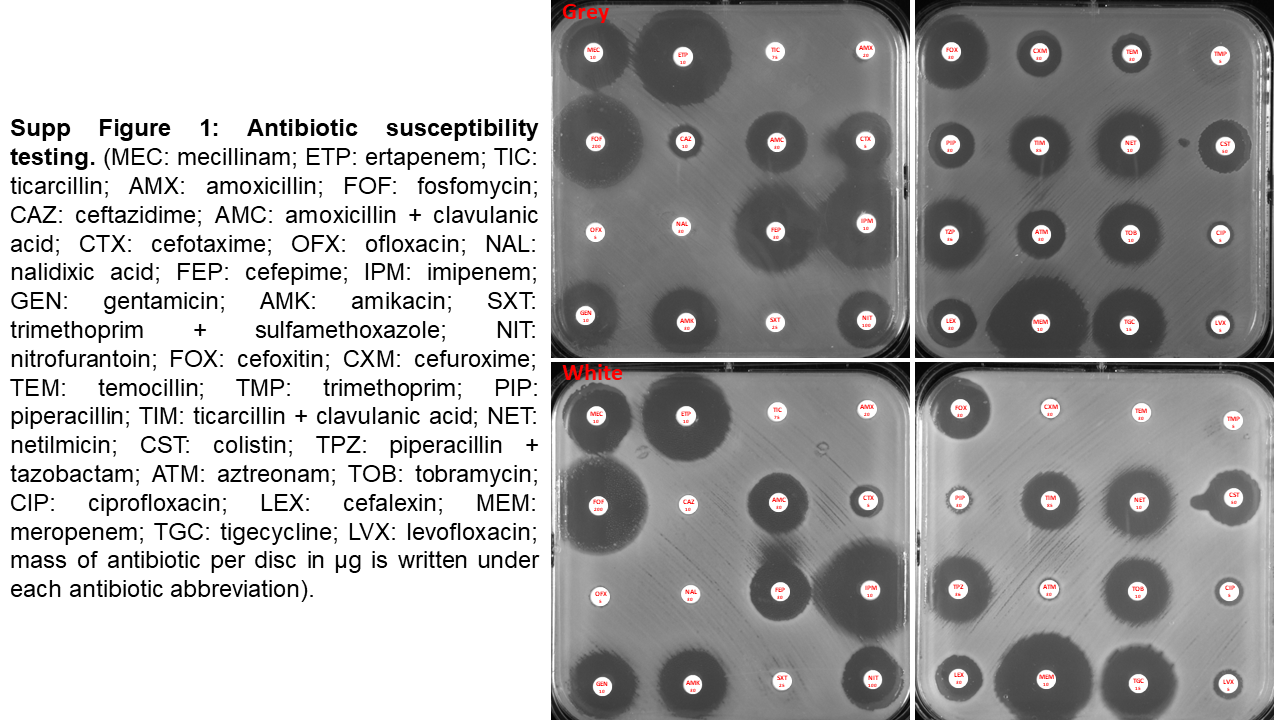

Supplement: Fig. S1 — Antibiotic susceptibility testing. [file aac.01459-23-s0001.tif]

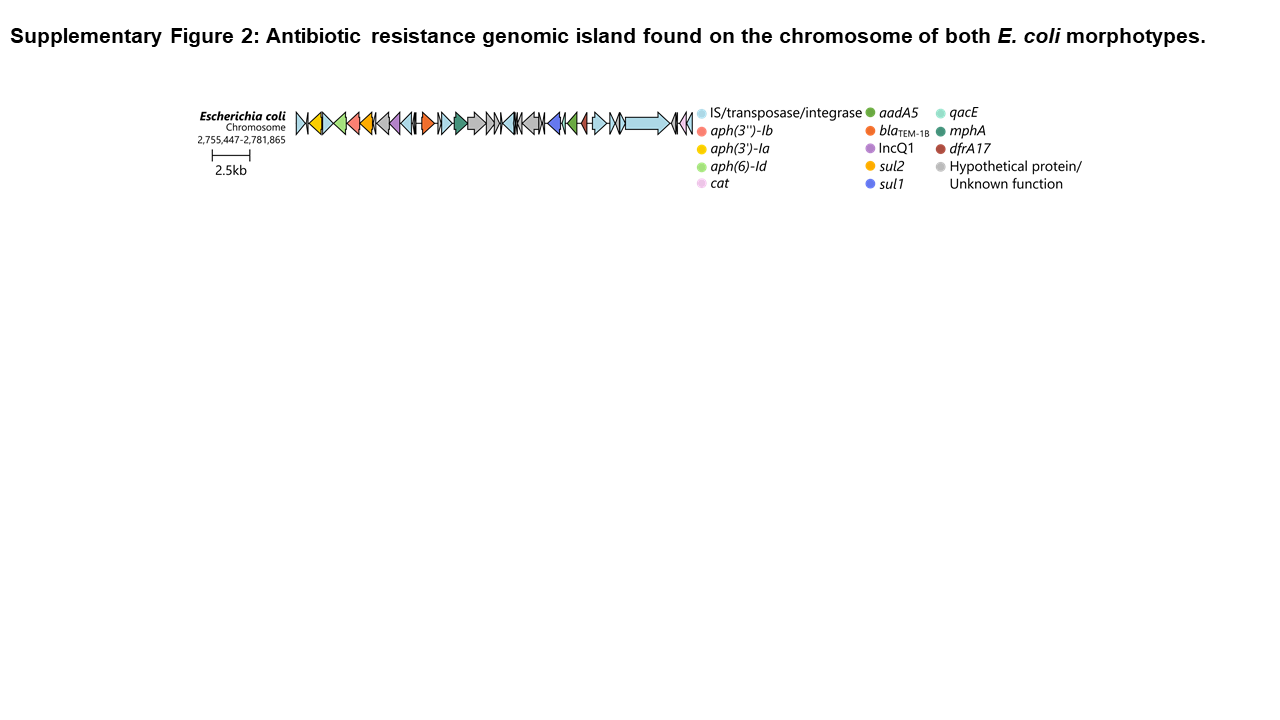

Supplement: Fig. S2 — Antibiotic resistance genomic island found on the chromosome of the E. coli morphotypes. [file aac.01459-23-s0002.tif]
