# Supplementary material for: Inter-phylum circulation of a beta-lactamase-encoding gene: a rare but observable event
Source: Antimicrob Agents Chemother. 2024 Mar 5;68(4):e01459-23. doi: 10.1128/aac.01459-23 (PMC10989005; doi:10.1128/aac.01459-23)
Supplement: Additional experimental details — Additional details about beta-lactamase characterization. [file aac.01459-23-s0003.pdf]

Supplementary materials:

Cloning and recombinant protein expression

The $bla_{MUN\text{-}1}$ gene was cloned into pTOPO-kanR vector using the pCR-Blunt TOPO cloning kit (Invitrogen) using specific primers (MUN Fw : 5'-GAG-CAT-CCG-CTT-CTT-TGG-GC-3' and MUN Rv : 5'-ACG-CAC-ACT-TTT-CCG-ATA-TG-3') spanning the full gene in order to express the whole protein. The resulting recombinant plasmid was transformed by heat shock into *E. coli* TOP10 (pTOPO/$bla_{MUN\text{-}1}$).

Purification of the protein

Purification of the MUN-1 beta-lactamase was carried out by ion-exchange chromatography. *E. coli* TOP10 (pTOPO/$bla_{MUN\text{-}1}$) was grown in 2 L of lysogeny broth (LB) containing 100 µg/mL of ampicillin, 50 µg/mL of kanamycin and 1 mM of IPTG. The overnight culture was centrifuged, and the obtained pellet was re-suspended in phosphate buffer pH 7 (0.1 M) and sonicated using a Vibra cellTM 75,186 sonicator (Thermo Fisher Scientific). After filtration using a 0.22 µm nitrocellulose filter, the crude extract was loaded in a prequilibrated SP-Sepharose column connected to an ÄktaPrime chromatography system (GE Healthcare) and eluted with a linear pH gradient using bis-Tris buffer at pH 6.4 (20 mM) and eluted with a linear NaCl gradient. The beta-lactamase recovered in the flow through was subsequently dialyzed against MES buffer at pH 6.4 (50 mM), loaded onto a Q-Sepharose column pre-equilibrated with the same buffer, and eluted with a linear NaCl gradient. The fractions containing the highest beta-lactamase activity, as determined by nitrocefin test (TRC Toronto, Canada), were pooled and dialyzed overnight against sodium phosphate buffer (pH 7, 0.1 M). The protein concentrations were measured using Bradford reagent (Sigma-Aldrich). The total protein content was measured by using a Bradford assay.

Determination of beta-lactamase relative molecular mass

The relative purity of beta-lactamase was estimated by SDS-12% PAGE (GeneScript) analysis. Enzyme extracts were boiled for 10 min in a 1%SDS-3% beta-mercaptoethanol solution (GenScript) and then were subjected to electrophoresis with marker (GenScript) at room temperature.

Enzymatic characterization

Purified beta-lactamase was used for kinetic measurements performed at room temperature in 100 mM sodium phosphate (pH 7.0). The initial rates of hydrolysis were determined with a Genesys 10S UV-visible spectrophotometer (Thermo Scientific). The following wavelengths and absorption coefficients were used: benzylpenicillin, 232 nm and ∆ε of -1,100 M-1 cm-1; ampicillin, 240 nm and ∆ε of -999 M-1 cm-1; ticarcillin, 235 nm and ∆ε of -1,050 M-1 cm-1; piperacillin, 235 nm and ∆ε of -1,070 M-1 cm-1; cephalothin, 262 nm and ∆ε of -7,960 M-1 cm-1 , cefoxitin, 265 nm and ∆ε of -7,380 M-1 cm-1; ceftazidime, 260 nm and ∆ε of -8,660 M-1 cm-1; cefepime, 264 nm and ∆ε of -8240 M-1 cm-1, cefotaxime, 265 nm and ∆ε of -6260 M-1 cm-1; imipenem, 297 nm and ∆ε of -9210 M-1 cm-1; meropenem, 297 nm and ∆ε of -9210 M-1 cm-1; aztreonam 318 nm and ∆ε of -640 M-1 cm-1. The *Ki* values were determined by direct competition assays using 100 M nitrocefin. Inverse initial steady-state velocities (1/*V*0) were plotted against the inhibitor concentration ([I]) to obtain a straight line. The plots were linear and provided *y* intercept and slope values used for *Ki* determinations. *Ki* was determined by dividing the value for the *y* intercept by the slope of the line and then was corrected by taking into account the cephalothin affinity, using the following equation: *Ki* (corrected) = *Ki* (observed)/(1 [S]/*Km*), where [S] is the concentration of nitrocefin (100 µM) used in the assay and *Km* is the Michaelis constant determined for nitrocefin (45.5 µM).

Susceptibility to beta-lactamase inhibitors

IC50 values were determined for clavulanic acid, tazobactam, and avibactam. Various concentrations of these inhibitors were pre-incubated with the purified enzyme MUN-1 during 3 min at room temperature, to determine the concentrations that reduced the hydrolysis rate with 100 M cephalothin by 50%. The results are expressed in nanomolar units.
