# Supplementary material for: Inter-phylum circulation of a beta-lactamase-encoding gene: a rare but observable event
Source: Antimicrob Agents Chemother. 2024 Mar 5;68(4):e01459-23. doi: 10.1128/aac.01459-23 (PMC10989005; doi:10.1128/aac.01459-23)
Supplement: Table S1 — Summary of assemblies obtained using Unicycler for white and gray E. coli morphotypes. [file aac.01459-23-s0004.pdf]

Supplementary Table 1: Summary of assemblies obtained using Unicycler for white and grey *E. coli* morphotypes.

	Chromosome		incFII		p0111	
	size (bp)	*n* *bla* $_{MUN-1}$	size (bp)	*n* *bla* $_{MUN-1}$	size (bp)	*n* *bla* $_{MUN-1}$
White	4,762,657	2	60,123	0	127,245	2
Grey	4,764,212	2	60,123	0	-	-
