# Supplementary material for: Inter-phylum circulation of a beta-lactamase-encoding gene: a rare but observable event
Source: Antimicrob Agents Chemother. 2024 Mar 5;68(4):e01459-23. doi: 10.1128/aac.01459-23 (PMC10989005; doi:10.1128/aac.01459-23)
Supplement: Table S2 — Antibiotic resistance genes (ARGs) found in the chromosome of both E. coli morphotypes using ResFinder (v4.0). [file aac.01459-23-s0005.pdf]

Supplementary Table 2: Antibiotic resistance genes (ARGs) found in the chromosome of both *E. coli* morphotypes using ResFinder (v4.0).

ARG Family	ARG name	Description	Resistance to	Identity	Coverage	Access number
ANT	*aadA5*	ANT(3")-Ia family aminoglycoside nucleotidyltransferase AadA5	Aminoglycosides	100.0	99.6	NG_047357.1.1
APH	*aph(3")-Ib*	aminoglycoside O-phosphotransferase APH(3")-Ib	Aminoglycosides	100.0	99.6	NG_047413.1.1
APH	*aph(6)-Id*	aminoglycoside O-phosphotransferase APH(6)-Id	Aminoglycosides	100.0	99.6	NG_047464.1.1
APH	*aph(3')-Ia*	aminoglycoside O-phosphotransferase APH(3')-Ia	Aminoglycosides	100.0	99.6	NG_047430.1.1
Cat	*catA1*	type A-1 chloramphenicol O-acetyltransferase	Chloramphenicol	99.3	65.9	NG_051704.1.1
Class A beta-lactamase	bla_{TEM-1}	class A broad-spectrum beta-lactamase TEM-1	Beta-lactams	100.0	99.7	NG_050145.1.1
Class C beta-lactamase	bla_{EC}	BlaEC family class C beta-lactamase	Beta-lactams	100.0	99.7	NG_047494.1.1
Dfr	*dfrA17*	trimethoprim-resistant dihydrofolate reductase DfrA17	Trimethoprim	99.4	99.4	NG_047709.1.1
Mph	*mph(A)*	Mph(A) family macrolide 2'-phosphotransferase	Macrolides	100.0	99.7	NG_047985.1.1
Sul	*sul1*	sulfonamide-resistant dihydropteroate synthase Sul1	Sulphonamides	99.6	99.6	NG_048081.1.1
Sul	*sul2*	Sulfonamide-resistant dihydropteroate synthase Sul2	Sulphonamides	100.0	99.6	NG_048118.1.1
Tet efflux	*tet(B)*	tetracycline efflux MFS transporter Tet(B)	Tetracyclines	100.0	99.8	NG_048163.1.1
