# Supplementary material for: Inter-phylum circulation of a beta-lactamase-encoding gene: a rare but observable event
Source: Antimicrob Agents Chemother. 2024 Mar 5;68(4):e01459-23. doi: 10.1128/aac.01459-23 (PMC10989005; doi:10.1128/aac.01459-23)
Supplement: Table S3 — ABRicate analysis of the virulence gene found in the genome of the E. coli morphotypes. [file aac.01459-23-s0006.pdf]

Supplementary Table 3: ABRicate analysis of the virulence gene found in the genome of both *E. coli* morphotypes.

Strain	SEQUENCE	START	END	STRAND	GENE	COVERAGE	COVERAGE MAP	GAPS	%COVERAGE	%IDENTITY	DATABASE	ACCESSION	PRODUCT	RESISTANCE
E. coli *bla* $_{MUN-1}$	1	6046	6149	-	entD	11-114/771	===...../......	2/2	13.36	90.48	Septicoli	NP_752599	(entD) phosphopantetheinyl transferase component of enterobactin synthase multienzyme complex [Enterobactin (VF0228)] [Escherichia coli CFT073]---iron acquisition	
E. coli *bla* $_{MUN-1}$	1	142517	142635	+	entD	1-119/771	===...../......	2/2	15.30	88.33	Septicoli	NP_752599	(entD) phosphopantetheinyl transferase component of enterobactin synthase multienzyme complex [Enterobactin (VF0228)] [Escherichia coli CFT073]---iron acquisition	
E. coli *bla* $_{MUN-1}$	1	177510	177645	-	entD	1-136/771	===...../......	2/2	17.51	86.86	Septicoli	NP_752599	(entD) phosphopantetheinyl transferase component of enterobactin synthase multienzyme complex [Enterobactin (VF0228)] [Escherichia coli CFT073]---iron acquisition	
E. coli *bla* $_{MUN-1}$	1	207361	207472	+	entD	1-113/771	===...../......	1/1	14.53	86.73	Septicoli	NP_752599	(entD) phosphopantetheinyl transferase component of enterobactin synthase multienzyme complex [Enterobactin (VF0228)] [Escherichia coli CFT073]---iron acquisition	
E. coli *bla* $_{MUN-1}$	1	216013	217413	+	gad_17	1-1401/1401	==============	0/0	100.00	99.86	Septicoli	U00096	Glutamate decarboxylase---miscellanous	
E. coli *bla* $_{MUN-1}$	1	243064	243200	-	entD	1-137/771	===...../......	2/2	17.64	90.58	Septicoli	NP_752599	(entD) phosphopantetheinyl transferase component of enterobactin synthase multienzyme complex [Enterobactin (VF0228)] [Escherichia coli CFT073]---iron acquisition	
E. coli *bla* $_{MUN-1}$	1	283895	284017	+	entD	11-132/771	===...../......	3/3	15.69	81.45	Septicoli	NP_752599	(entD) phosphopantetheinyl transferase component of enterobactin synthase multienzyme complex [Enterobactin (VF0228)] [Escherichia coli CFT073]---iron acquisition	
E. coli *bla* $_{MUN-1}$	1	289706	289836	-	entD	11-140/771	===...../......	2/3	16.73	83.33	Septicoli	NP_752599	(entD) phosphopantetheinyl transferase component of enterobactin synthase multienzyme complex [Enterobactin (VF0228)] [Escherichia coli CFT073]---iron acquisition	
E. coli *bla* $_{MUN-1}$	1	317924	318060	+	entD	1-137/771	===...../......	2/2	17.64	89.13	Septicoli	NP_752599	(entD) phosphopantetheinyl transferase component of enterobactin synthase multienzyme complex [Enterobactin (VF0228)] [Escherichia coli CFT073]---iron acquisition	
E. coli *bla* $_{MUN-1}$	1	369005	369131	+	entD	2-136/771	===...../......	2/8	16.47	82.96	Septicoli	NP_752599	(entD) phosphopantetheinyl transferase component of enterobactin synthase multienzyme complex [Enterobactin (VF0228)] [Escherichia coli CFT073]---iron acquisition	
E. coli *bla* $_{MUN-1}$	1	549755	549887	-	entD	1-132/771	===...../......	3/3	16.99	86.57	Septicoli	NP_752599	(entD) phosphopantetheinyl transferase component of enterobactin synthase multienzyme complex [Enterobactin (VF0228)] [Escherichia coli CFT073]---iron acquisition	
E. coli *bla* $_{MUN-1}$	1	612803	612942	+	entD	1-140/771	===...../......	2/2	18.03	86.53	Septicoli	NP_752599	(entD) phosphopantetheinyl transferase component of enterobactin synthase multienzyme complex [Enterobactin (VF0228)] [Escherichia coli CFT073]---iron acquisition	
E. coli *bla* $_{MUN-1}$	1	630451	630575	-	entD	1-126/771	===...../......	2/3	16.08	81.10	Septicoli	NP_752599	(entD) phosphopantetheinyl transferase component of enterobactin synthase multienzyme complex [Enterobactin (VF0228)] [Escherichia coli CFT073]---iron acquisition	
E. coli *bla* $_{MUN-1}$	1	791021	791145	-	entD	1-126/771	===...../......	3/3	16.08	90.55	Septicoli	NP_752599	(entD) phosphopantetheinyl transferase component of enterobactin synthase multienzyme complex [Enterobactin (VF0228)] [Escherichia coli CFT073]---iron acquisition	
E. coli *bla* $_{MUN-1}$	1	848556	848692	-	entD	1-137/771	===...../......	3/4	17.51	85.61	Septicoli	NP_752599	(entD) phosphopantetheinyl transferase component of enterobactin synthase multienzyme complex [Enterobactin (VF0228)] [Escherichia coli CFT073]---iron acquisition	
E. coli *bla* $_{MUN-1}$	1	922631	922754	+	entD	1-125/771	===...../......	2/3	15.95	89.68	Septicoli	NP_752599	(entD) phosphopantetheinyl transferase component of enterobactin synthase multienzyme complex [Enterobactin (VF0228)] [Escherichia coli CFT073]---iron acquisition	
E. coli *bla* $_{MUN-1}$	1	950629	950757	-	entD	10-140/771	===...../......	2/2	16.73	83.97	Septicoli	NP_752599	(entD) phosphopantetheinyl transferase component of enterobactin synthase multienzyme complex [Enterobactin (VF0228)] [Escherichia coli CFT073]---iron acquisition	
E. coli *bla* $_{MUN-1}$	1	1E+06	1E+06	+	entD	1-142/771	===...../......	3/3	18.16	90.21	Septicoli	NP_752599	(entD) phosphopantetheinyl transferase component of enterobactin synthase multienzyme complex [Enterobactin (VF0228)] [Escherichia coli CFT073]---iron acquisition	
E. coli *bla* $_{MUN-1}$	1	1E+06	1E+06	+	entD	1-133/771	===...../......	2/2	17.12	88.81	Septicoli	NP_752599	(entD) phosphopantetheinyl transferase component of enterobactin synthase multienzyme complex [Enterobactin (VF0228)] [Escherichia coli CFT073]---iron acquisition	
E. coli *bla* $_{MUN-1}$	1	1E+06	1E+06	+	entD	14-122/771	===...../......	3/5	13.62	84.55	Septicoli	NP_752599	(entD) phosphopantetheinyl transferase component of enterobactin synthase multienzyme complex [Enterobactin (VF0228)] [Escherichia coli CFT073]---iron acquisition	
E. coli *bla* $_{MUN-1}$	1	1E+06	1E+06	+	entD	1-137/771	===...../......	2/2	17.64	90.58	Septicoli	NP_752599	(entD) phosphopantetheinyl transferase component of enterobactin synthase multienzyme complex [Enterobactin (VF0228)] [Escherichia coli CFT073]---iron acquisition	
E. coli *bla* $_{MUN-1}$	1	2E+06	2E+06	+	entD	1-137/771	===...../......	2/2	17.64	89.86	Septicoli	NP_752599	(entD) phosphopantetheinyl transferase component of enterobactin synthase multienzyme complex [Enterobactin (VF0228)] [Escherichia coli CFT073]---iron acquisition	
E. coli *bla* $_{MUN-1}$	1	2E+06	2E+06	-	entD	15-137/771	===...../......	3/4	15.69	84.00	Septicoli	NP_752599	(entD) phosphopantetheinyl transferase component of enterobactin synthase multienzyme complex [Enterobactin (VF0228)] [Escherichia coli CFT073]---iron acquisition	
E. coli *bla* $_{MUN-1}$	1	2E+06	2E+06	-	entD	15-126/771	===...../......	2/2	14.40	92.03	Septicoli	NP_752599	(entD) phosphopantetheinyl transferase component of enterobactin synthase multienzyme complex [Enterobactin (VF0228)] [Escherichia coli CFT073]---iron acquisition	
E. coli *bla* $_{MUN-1}$	1	2E+06	2E+06	-	entD	1-137/771	===...../......	2/2	17.64	91.30	Septicoli	NP_752599	(entD) phosphopantetheinyl transferase component of enterobactin synthase multienzyme complex [Enterobactin (VF0228)] [Escherichia coli CFT073]---iron acquisition	
E. coli *bla* $_{MUN-1}$	1	2E+06	2E+06	-	espL1	1-1899/1899	========/======	1/1	100.00	97.58	Septicoli	NP_288154	(espL1) Type III secretion system effector espL1 [LEE encoded T3SS (SS020)] [Escherichia coli O157:H7 str. EDL933]---toxin	
E. coli *bla* $_{MUN-1}$	1	2E+06	2E+06	-	malX	1-1593/1593	==============	0/0	100.00	97.93	Septicoli	CP000247:1618614-1620206	PTS system maltose-specific EIICB component---miscellanous	
E. coli *bla* $_{MUN-1}$	1	2E+06	2E+06	+	entD	1-127/771	===...../......	1/1	16.34	83.47	Septicoli	NP_752599	(entD) phosphopantetheinyl transferase component of enterobactin synthase multienzyme complex [Enterobactin (VF0228)] [Escherichia coli CFT073]---iron acquisition	
E. coli *bla* $_{MUN-1}$	1	2E+06	2E+06	+	gad_27	1-1401/1401	==============	0/0	100.00	100.00	Septicoli	U00096	Glutamate decarboxylase---miscellanous	
E. coli *bla* $_{MUN-1}$	1	2E+06	2E+06	-	entD	10-145/771	===...../......	4/5	17.12	86.13	Septicoli	NP_752599	(entD) phosphopantetheinyl transferase component of enterobactin synthase multienzyme complex [Enterobactin (VF0228)] [Escherichia coli CFT073]---iron acquisition	
E. coli *bla* $_{MUN-1}$	1	2E+06	2E+06	-	entD	10-141/771	===...../......	2/3	16.86	85.71	Septicoli	NP_752599	(entD) phosphopantetheinyl transferase component of enterobactin synthase multienzyme complex [Enterobactin (VF0228)] [Escherichia coli CFT073]---iron acquisition	
E. coli *bla* $_{MUN-1}$	1	2E+06	2E+06	+	espR1	198-490/1260	..====.........	0/0	23.25	94.20	Septicoli	NP_287686	(espR1) Type III secretion system effector espR1 [LEE encoded T3SS (SS020)] [Escherichia coli O157:H7 str. EDL933]---toxin	
E. coli *bla* $_{MUN-1}$	1	2E+06	2E+06	+	espR1	474-1253/1260	.....===/======	2/2	61.75	93.33	Septicoli	NP_287686	(espR1) Type III secretion system effector espR1 [LEE encoded T3SS (SS020)] [Escherichia coli O157:H7 str. EDL933]---toxin	
E. coli *bla* $_{MUN-1}$	1	3E+06	3E+06	+	entD	3-114/771	===...../......	2/2	14.40	87.61	Septicoli	NP_752599	(entD) phosphopantetheinyl transferase component of enterobactin synthase multienzyme complex [Enterobactin (VF0228)] [Escherichia coli CFT073]---iron acquisition	
E. coli *bla* $_{MUN-1}$	1	3E+06	3E+06	+	entD	1-132/771	===...../......	2/2	16.99	82.71	Septicoli	NP_752599	(entD) phosphopantetheinyl transferase component of enterobactin synthase multienzyme complex [Enterobactin (VF0228)] [Escherichia coli CFT073]---iron acquisition	
E. coli *bla* $_{MUN-1}$	1	3E+06	3E+06	+	ompA	1-1041/1041	==============	0/0	100.00	97.98	Septicoli	AAF37887	(ompA) outer membrane protein A [OmpA (VF0236)] [Escherichia coli O18:K1:H7 str. RS218]---protectin	
E. coli *bla* $_{MUN-1}$	1	3E+06	3E+06	-	entD	1-137/771	===...../......	2/2	17.64	87.68	Septicoli	NP_752599	(entD) phosphopantetheinyl transferase component of enterobactin synthase multienzyme complex [Enterobactin (VF0228)] [Escherichia coli CFT073]---iron acquisition	
E. coli *bla* $_{MUN-1}$	1	3E+06	3E+06	-	entD	1-137/771	===...../......	2/2	17.64	89.13	Septicoli	NP_752599	(entD) phosphopantetheinyl transferase component of enterobactin synthase multienzyme complex [Enterobactin (VF0228)] [Escherichia coli CFT073]---iron acquisition	
E. coli *bla* $_{MUN-1}$	1	3E+06	3E+06	+	entD	2-132/771	===...../......	2/2	16.86	87.88	Septicoli	NP_752599	(entD) phosphopantetheinyl transferase component of enterobactin synthase multienzyme complex [Enterobactin (VF0228)] [Escherichia coli CFT073]---iron acquisition	
E. coli *bla* $_{MUN-1}$	1	3E+06	3E+06	+	entD	14-132/771	===...../......	3/3	15.30	92.56	Septicoli	NP_752599	(entD) phosphopantetheinyl transferase component of enterobactin synthase multienzyme complex [Enterobactin (VF0228)] [Escherichia coli CFT073]---iron acquisition	
E. coli *bla* $_{MUN-1}$	1	3E+06	3E+06	+	entD	1-136/771	===...../......	2/3	17.38	88.32	Septicoli	NP_752599	(entD) phosphopantetheinyl transferase component of enterobactin synthase multienzyme complex [Enterobactin (VF0228)] [Escherichia coli CFT073]---iron acquisition	
E. coli *bla* $_{MUN-1}$	1	3E+06	3E+06	-	entD	3-137/771	===...../......	3/3	17.38	81.75	Septicoli	NP_752599	(entD) phosphopantetheinyl transferase component of enterobactin synthase multienzyme complex [Enterobactin (VF0228)] [Escherichia coli CFT073]---iron acquisition	
E. coli *bla* $_{MUN-1}$	1	3E+06	3E+06	+	entD	6-130/771	===...../......	2/2	16.08	87.30	Septicoli	NP_752599	(entD) phosphopantetheinyl transferase component of enterobactin synthase multienzyme complex [Enterobactin (VF0228)] [Escherichia coli CFT073]---iron acquisition	
E. coli *bla* $_{MUN-1}$	1	3E+06	3E+06	+	entD	11-126/771	===...../......	2/2	14.92	89.74	Septicoli	NP_752599	(entD) phosphopantetheinyl transferase component of enterobactin synthase multienzyme complex [Enterobactin (VF0228)] [Escherichia coli CFT073]---iron acquisition	
E. coli *bla* $_{MUN-1}$	1	3E+06	3E+06	-	entD	1-132/771	===...../......	2/2	16.99	90.98	Septicoli	NP_752599	(entD) phosphopantetheinyl transferase component of enterobactin synthase multienzyme complex [Enterobactin (VF0228)] [Escherichia coli CFT073]---iron acquisition	
E. coli *bla* $_{MUN-1}$	1	3E+06	3E+06	-	entA	1-747/747	==============	0/0	100.00	95.72	Septicoli	NP_752614	(entA) 23-dihydro-23-dihydroxybenzoate dehydrogenase [Enterobactin (VF0228)] [Escherichia coli CFT073]---iron acquisition	
E. coli *bla* $_{MUN-1}$	1	3E+06	3E+06	-	entB	1-858/858	==============	0/0	100.00	98.14	Septicoli	NP_752613	(entB) isochorismatase [Enterobactin (VF0228)] [Escherichia coli CFT073]---iron acquisition	
E. coli *bla* $_{MUN-1}$	1	3E+06	3E+06	-	entE	1-1611/1611	==============	0/0	100.00	95.47	Septicoli	NP_752612	(entE) 23-dihydroxybenzoate-AMP ligase component of enterobactin synthase multienzyme complex [Enterobactin (VF0228)] [Escherichia coli CFT073]---iron acquisition	
E. coli *bla* $_{MUN-1}$	1	3E+06	3E+06	-	entC	1-1188/1188	==============	0/0	100.00	97.90	Septicoli	NP_752611	(entC) isochorismate synthase 1 [Enterobactin (VF0228)] [Escherichia coli CFT073]---iron acquisition	
E. coli *bla* $_{MUN-1}$	1	3E+06	3E+06	+	fepB	1-957/957	==============	0/0	100.00	97.49	Septicoli	NP_752610	(fepB) ferrienterobactin ABC transporter periplasmic binding protein [Enterobactin (VF0228)] [Escherichia coli CFT073]---iron acquisition	
E. coli *bla* $_{MUN-1}$	1	3E+06	3E+06	-	entS	1-1251/1251	==============	0/0	100.00	95.44	Septicoli	NP_752609	(entS) enterobactin exporter iron-regulated [enterobactin (IA019)] [Escherichia coli CFT073]---iron acquisition	
E. coli *bla* $_{MUN-1}$	1	3E+06	3E+06	+	fepD	1-1017/1017	==============	0/0	100.00	96.07	Septicoli	NP_752608	(fepD) ferrienterobactin ABC transporter permease [Enterobactin (VF0228)] [Escherichia coli CFT073]---iron acquisition	
E. coli *bla* $_{MUN-1}$	1	3E+06	3E+06	+	fepG	1-993/993	==============	0/0	100.00	94.06	Septicoli	NP_752607	(fepG) iron-enterobactin ABC transporter permease [Enterobactin (VF0228)] [Escherichia coli CFT073]---iron acquisition	
E. coli *bla* $_{MUN-1}$	1	3E+06	3E+06	+	fepC	1-816/816	==============	0/0	100.00	97.18	Septicoli	NP_752606	(fepC) ferrienterobactin ABC transporter ATPase [Enterobactin (VF0228)] [Escherichia coli CFT073]---iron acquisition	
E. coli *bla* $_{MUN-1}$	1	3E+06	3E+06	-	entF	1-3854/3882	==============	0/0	99.28	95.69	Septicoli	NP_752604	(entF) enterobactin synthase multienzyme complex component ATP-dependent [Enterobactin (VF0228)] [Escherichia coli CFT073]---iron acquisition	
E. coli *bla* $_{MUN-1}$	1	3E+06	3E+06	-	fes	1-1203/1203	==============	0/0	100.00	96.67	Septicoli	NP_752602	(fes) enterobactin/ferric enterobactin esterase [enterobactin (IA019)] [Escherichia coli CFT073]---iron acquisition	
E. coli *bla* $_{MUN-1}$	1	3E+06	3E+06	+	fepA	1-2241/2241	==============	0/0	100.00	96.74	Septicoli	NP_752600	(fepA) ferrienterobactin outer membrane transporter [Enterobactin (VF0228)] [Escherichia coli CFT073]---iron acquisition	
E. coli *bla* $_{MUN-1}$	1	3E+06	3E+06	+	entD	1-771/771	==============	0/0	100.00	95.20	Septicoli	NP_752599	(entD) phosphopantetheinyl transferase component of enterobactin synthase multienzyme complex [Enterobactin (VF0228)] [Escherichia coli CFT073]---iron acquisition	
E. coli *bla* $_{MUN-1}$	1	4E+06	4E+06	-	entD	1-126/771	===...../......	2/2	16.21	88.98	Septicoli	NP_752599	(entD) phosphopantetheinyl transferase component of enterobactin synthase multienzyme complex [Enterobactin (VF0228)] [Escherichia coli CFT073]---iron acquisition	
E. coli *bla* $_{MUN-1}$	1	4E+06	4E+06	+	entD	1-136/771	===...../......	4/4	17.38	83.33	Septicoli	NP_752599	(entD) phosphopantetheinyl transferase component of enterobactin synthase multienzyme complex [Enterobactin (VF0228)] [Escherichia coli CFT073]---iron acquisition	
E. coli *bla* $_{MUN-1}$	1	4E+06	4E+06	-	fdeC	1-4246/4251	========/======	1/3	99.88	94.23	Septicoli	YP_002390132	(fdeC) adhesin FdeC [FdeC (VF0506)] [Escherichia coli O45:K1:H7 str. S88]---adhesin	
E. coli *bla* $_{MUN-1}$	1	4E+06	4E+06	+	ykgK/ecpR	1-591/591	==============	0/0	100.00	96.95	Septicoli	NP_286011	(ykgK/ecpR) regulator protein EcpR [ECP (VF0404)] [Escherichia coli O157:H7 str. EDL933]---adhesin	
E. coli *bla* $_{MUN-1}$	1	4E+06	4E+06	+	yagZ/ecpA	1-588/588	==============	0/0	100.00	99.15	Septicoli	NP_286010	(yagZ/ecpA) E. coli common pilus structural subunit EcpA [ECP (VF0404)] [Escherichia coli O157:H7 str. EDL933]---adhesin	
E. coli *bla* $_{MUN-1}$	1	4E+06	4E+06	+	yagY/ecpB	1-669/669	==============	0/0	100.00	98.06	Septicoli	NP_286009	(yagY/ecpB) E. coli common pilus chaperone EcpB [ECP (VF0404)] [Escherichia coli O157:H7 str. EDL933]---adhesin	
E. coli *bla* $_{MUN-1}$	1	4E+06	4E+06	+	yagX/ecpC	1-2526/2526	==============	0/0	100.00	98.77	Septicoli	NP_286008	(yagX/ecpC) E. coli common pilus usher EcpC [ECP (VF0404)] [Escherichia coli O157:H7 str. EDL933]---adhesin	
E. coli *bla* $_{MUN-1}$	1	4E+06	4E+06	+	yagW/ecpD	1-1644/1644	==============	0/0	100.00	99.09	Septicoli	NP_286007	(yagW/ecpD) polymerized tip adhesin of ECP fibers [ECP (VF0404)] [Escherichia coli O157:H7 str. EDL933]---adhesin	
E. coli *bla* $_{MUN-1}$	1	4E+06	4E+06	+	yagV/ecpE	1-754/756	==============	0/0	99.74	97.08	Septicoli	NP_286006	(yagV/ecpE) E. coli common pilus chaperone EcpE [ECP (VF0404)] [Escherichia coli O157:H7 str. EDL933]---adhesin	
E. coli *bla* $_{MUN-1}$	1	4E+06	4E+06	+	entD	1-132/771	===...../......	2/2	16.99	88.72	Septicoli	NP_752599	(entD) phosphopantetheinyl transferase component of enterobactin synthase multienzyme complex [Enterobactin (VF0228)] [Escherichia coli CFT073]---iron acquisition	
E. coli *bla* $_{MUN-1}$	1	4E+06	4E+06	-	espY1	1-753/753	========/======	3/61	99.87	86.96	Septicoli	NP_285753	(espY1) Type III secretion system effector EspY1 [LEE encoded T3SS (SS020)] [Escherichia coli O157:H7 str. EDL933]---toxin	
E. coli *bla* $_{MUN-1}$	1	4E+06	4E+06	+	espX1	1-1422/1422	==============	0/0	100.00	93.25	Septicoli	NP_285716	(espX1) Type III secretion system effector EspX1 [LEE encoded T3SS (SS020)] [Escherichia coli O157:H7 str. EDL933]---toxin	
E. coli *bla* $_{MUN-1}$	1	4E+06	4E+06	+	entD	14-98/771	==....../......	2/2	10.89	90.70	Septicoli	NP_752599	(entD) phosphopantetheinyl transferase component of enterobactin synthase multienzyme complex [Enterobactin (VF0228)] [Escherichia coli CFT073]---iron acquisition	
E. coli *bla* $_{MUN-1}$	1	4E+06	4E+06	-	fimH	1-912/912	==============	0/0	100.00	97.37	Septicoli	NP_757248	(fimH) FimH protein precursor [Type 1 fimbriae (VF0221)] [Escherichia coli CFT073]---adhesin	
E. coli *bla* $_{MUN-1}$	1	4E+06	4E+06	-	fimG	1-504/504	==============	0/0	100.00	97.82	Septicoli	NP_757247	(fimG) FimG protein precursor [Type 1 fimbriae (VF0221)] [Escherichia coli CFT073]---adhesin	
E. coli *bla* $_{MUN-1}$	1	4E+06	4E+06	-	fimF	1-534/534	==============	0/0	100.00	97.94	Septicoli	NP_757245	(fimF) FimF protein precursor [Type 1 fimbriae (VF0221)] [Escherichia coli CFT073]---adhesin	
E. coli *bla* $_{MUN-1}$	1	4E+06	4E+06	-	fimD	1-2637/2637	==============	0/0	100.00	98.60	Septicoli	NP_757244	(fimD) Outer membrane usher protein fimD precursor [Type 1 fimbriae (VF0221)] [Escherichia coli CFT073]---adhesin	
E. coli *bla* $_{MUN-1}$	1	4E+06	4E+06	-	fimC	1-726/726	==============	0/0	100.00	98.90	Septicoli	NP_757243	(fimC) Chaperone protein fimC precursor [Type 1 fimbriae (VF0221)] [Escherichia coli CFT073]---adhesin	
E. coli *bla* $_{MUN-1}$	1	4E+06	4E+06	-	fimI	1-540/540	==============	0/0	100.00	98.70	Septicoli	NP_757242	(fimI) Fimbrin-like protein fimI precursor [Type 1 fimbriae (VF0221)] [Escherichia coli CFT073]---adhesin	
E. coli *bla* $_{MUN-1}$	1	4E+06	4E+06	-	fimA	1-606/606	========/======	1/6	100.00	88.56	Septicoli	NP_757241	(fimA) Type-1 fimbrial protein A chain precursor [Type 1 fimbriae (VF0221)] [Escherichia coli CFT073]---adhesin	
E. coli *bla* $_{MUN-1}$	1	4E+06	4E+06	-	fimE	1-595/597	==============	0/0	99.66	99.33	Septicoli	NP_757240	(fimE) Type 1 fimbriae Regulatory protein fimE [Type 1 fimbriae (VF0221)] [Escherichia coli CFT073]---adhesin	
E. coli *bla* $_{MUN-1}$	1	4E+06	4E+06	-	fimB	1-603/603	==============	0/0	100.00	98.18	Septicoli	NP_757239	(fimB) Type 1 fimbriae Regulatory protein fimB [Type 1 fimbriae (VF0221)] [Escherichia coli CFT073]---adhesin	
E. coli *bla* $_{MUN-1}$	1	4E+06	4E+06	-	entD	1-132/771	===...../......	2/12	15.69	84.96	Septicoli	NP_752599	(entD) phosphopantetheinyl transferase component of enterobactin synthase multienzyme complex [Enterobactin (VF0228)] [Escherichia coli CFT073]---iron acquisition	
E. coli *bla* $_{MUN-1}$	1	4E+06	4E+06	+	entD	1-136/771	===...../......	2/2	17.51	83.21	Septicoli	NP_752599	(entD) phosphopantetheinyl transferase component of enterobactin synthase multienzyme complex [Enterobactin (VF0228)] [Escherichia coli CFT073]---iron acquisition	
E. coli *bla* $_{MUN-1}$	1	4E+06	4E+06	-	entD	2-114/771	===...../......	2/2	14.53	88.60	Septicoli	NP_752599	(entD) phosphopantetheinyl transferase component of enterobactin synthase multienzyme complex [Enterobactin (VF0228)] [Escherichia coli CFT073]---iron acquisition	
E. coli *bla* $_{MUN-1}$	1	4E+06	4E+06	+	entD	6-129/771	===...../......	2/3	15.95	81.75	Septicoli	NP_752599	(entD) phosphopantetheinyl transferase component of enterobactin synthase multienzyme complex [Enterobactin (VF0228)] [Escherichia coli CFT073]---iron acquisition	
E. coli *bla* $_{MUN-1}$	1	4E+06	4E+06	+	espX5	1-1293/1293	==============	0/0	100.00	97.14	Septicoli	NP_290699	(espX5) Type III secretion system effector EspX5 [LEE encoded T3SS (SS020)] [Escherichia coli O157:H7 str. EDL933]---toxin	
E. coli *bla* $_{MUN-1}$	1	4E+06	4E+06	-	espX4	126-1581/1581	.=======/======	2/2	92.03	93.75	Septicoli	NP_290672	(espX4) Type III secretion system effector EspX4 [LEE encoded T3SS (SS020)] [Escherichia coli O157:H7 str. EDL933]---toxin	
E. coli *bla* $_{MUN-1}$	1	4E+06	4E+06	-	espX4	1-134/1581	==............	0/0	8.48	88.06	Septicoli	NP_290672	(espX4) Type III secretion system effector EspX4 [LEE encoded T3SS (SS020)] [Escherichia coli O157:H7 str. EDL933]---toxin	
E. coli *bla* $_{MUN-1}$	1	4E+06	4E+06	+	espL4	1-2187/2187	==============	0/0	100.00	97.35	Septicoli	NP_290644	(espL4) Type III secretion system effector EspL4 [LEE encoded T3SS (SS020)] [Escherichia coli O157:H7 str. EDL933]---toxin	
E. coli *bla* $_{MUN-1}$	1	5E+06	5E+06	-	entD	15-124/771	===...../......	2/2	14.14	90.09	Septicoli	NP_752599	(entD) phosphopantetheinyl transferase component of enterobactin synthase multienzyme complex [Enterobactin (VF0228)] [Escherichia coli CFT073]---iron acquisition	
E. coli *bla* $_{MUN-1}$	1	5E+06	5E+06	-	entD	1-113/771	===...../......	1/1	14.53	88.50	Septicoli	NP_752599	(entD) phosphopantetheinyl transferase component of enterobactin synthase multienzyme complex [Enterobactin (VF0228)] [Escherichia coli CFT073]---iron acquisition	
E. coli *bla* $_{MUN-1}$	1	5E+06	5E+06	+	entD	1-131/771	===...../......	2/2	16.86	87.88	Septicoli	NP_752599	(entD) phosphopantetheinyl transferase component of enterobactin synthase multienzyme complex [Enterobactin (VF0228)] [Escherichia coli CFT073]---iron acquisition	
E. coli *bla* $_{MUN-1}$	1	5E+06	5E+06	-	entD	1-137/771	===...../......	2/2	17.64	84.78	Septicoli	NP_752599	(entD) phosphopantetheinyl transferase component of enterobactin synthase multienzyme complex [Enterobactin (VF0228)] [Escherichia coli CFT073]---iron acquisition	
E. coli *bla* $_{MUN-1}$	1	5E+06	5E+06	+	aslA	1-1428/1428	========/======	1/1	100.00	93.56	Septicoli	AAG10151	(aslA) putative arylsulfatase [AslA (VF0238)] [Escherichia coli O18:K1:H7 str. RS218]---invasin	
E. coli *bla* $_{MUN-1}$	3	46600	47331	+	traT	1-732/732	==============	0/0	100.00	98.09	Septicoli	J01769.1:403-1134	Plasmid R100 (from E.coli) complement resistance (traT) gene---protectin	
