# Supplementary material for: Inter-phylum circulation of a beta-lactamase-encoding gene: a rare but observable event
Source: Antimicrob Agents Chemother. 2024 Mar 5;68(4):e01459-23. doi: 10.1128/aac.01459-23 (PMC10989005; doi:10.1128/aac.01459-23)
Supplement: Table S4 — Hits obtained analyzing the blaMUN-1 gene distribution using BLASTN against RefSeq genomes database. [file aac.01459-23-s0007.pdf]

Supplementary Table 4: Hits obtained analyzing the *bla* $_{MUN-1}$ gene distribution using BLASTN against RefSeq genomes database (70% nucleotide identity, 80% coverage).

**Species**	**RefSeq Genomes entries**	**% identity**	**Query cover**	**Accession numbers**	**max copies /AN**	**% of contigs >500kb**
Alistipes shahii	31	100	100	NZ_JADMNV010000001.1	1	100
Alistipes putredinis	75	100	100	NZ_CAUFBO010000004.1	1	0
Bacteroides caccae	99	100	100	NZ_RCXH01000036.1; NZ_VVYI01000057.1; NZ_VVYJ01000032.1	1	0
Bacteroides eggerthii	54	100	100	NZ_RCXL01000057.1; NZ_VVZY01000116.1; NZ_VVZX01000033.1	1	0
Bacteroides fragilis	566	100	100	NZ_VOHY01000006.1; NZ_JANGCS010000017.1; NZ_JAJCKU010000030.1	1	0
Bacteroides ovatus	288	100	100	NZ_JAHYNG010000035.1; NZ_JAQEVX010000058.1	1	0
Bacteroides salyersiae	58	99.9-100	100	NZ_WCKY01000001.1; NZ_WCKW01000090.1; NZ_WCKX01000075.1; NZ_JABFHZ010000002.1; NZ_CP081902.1	1	20
Bacteroides stercoris	98	100	100	NZ_CABOGI010000053.1; NZ_JAQNVU010000011.1; NZ_QSSP01000053.1; NZ_QROW01000004.1; NZ_JAQNVT010000010.1	1	0
Bacteroides thetaiotaomicron	328	100	100	NZ_JAHYLL010000075.1; NZ_JAHYPH010000024.1; NZ_JADPBE010000005.1; NZ_JAQNVD010000005.1; NZ_JANUPG010000001.1	1	20
Bacteroides uniformis	365	100	100	NZ_WCUR01000119.1; NZ_WCUP01000009.1; NZ_WCUQ01000001.1; NZ_JANUJA010000001.1; NZ_AP019725.1; NZ_AP019724.1	6	17
Bacteroides sp.	53	100	100	NZ_JAPQKZ010000100.1; NZ_CP083673.1; NZ_CAAFEE010000290.1	1	33
Bacteroides xylanisolvens	195	99.9-100	100	NZ_WDEE01000003.1; NZ_WDEF01000003.1; NZ_JAHOJA010000006.1	1	0
Barnesiella propionica	1	71.9	88	NZ_JAOQJK010000002.1	1	100
Butyricimonas faecihominis	8	100	100	NZ_JAQEXV010000004.1	1	0
Odoribacter splanchnicus	52	100	100	NZ_JADNDE010000093.1; NZ_JADNIQ010000002.1	1	0
Parabacteroides distasonis	253	99.9-100	100	NZ_JAHONS010000002.1; NZ_JAHONT010000002.1; NZ_JAHYMP010000020.1; NZ_JAJCJX010000007.1; NZ_JAJCNE010000002.1; NZ_WKMM01000035.1; NZ_JAQEXU010000002.1; NZ_JAQMQB010000055.1; NZ_JAQMQC010000053.1; NZ_AP019729.1; NZ_BQOD01000001.1; NZ_BQOC01000001.1; NZ_JAHYMB010000033.1; NZ_CP103256.1	1	7
Parabacteroides faecis	5	100	100	NZ_JACRTM010000033.1	1	0
Parabacteroides goldsteinii	102	100	100	NZ_BQND01000001.1; NZ_BQNC01000001.1	2	50
Parabacteroides johnsonii	36	100	100	NZ_JAASIA010000002.1; NZ_JANUNE010000002.1; NZ_JANUNE010000003.1	3	33
Parabacteroides merdae	126	100	100	NZ_JADNHS010000020.1; NZ_JADMOA010000018.1; NZ_JAQDNL010000017.1; NZ_JAQMOI010000016.1; NZ_JAQMOJ010000018.1	1	0
Parabacteroides sp.				NZ_KQ236102.1; NZ_JAMOKM010000041.1; NZ_JAOEGA010000001.1; NZ_QTMV01000009.1	1	25
Leyella stercorea	49	99.9-100	100	NZ_JAIJUX010000025.1; NZ_QRNO01000005.1; NZ_CABJDY010000005.1	1	0
Paraprevotella clara	27	100	100	NZ_AP025941.1	2	100
Prevotellamassilia timonensis	3	100	100	NZ_LT629842.1; NZ_LT629840.1; NZ_LT629830.1; NZ_LT629845.1	1	25
Phocaeicola massiliensis	27	100	100	NZ_JADMTU010000014.1; NZ_JAQCSN010000016.1; NZ_JAQCTA010000014.1	1	0
Phocaeicola dorei	114	100	100	NZ_BQOB01000001.1; NZ_BQOA01000001.1; NZ_JAKNHN010000004.1; NZ_JADMOY010000018.1; NZ_JADNHW010000026.1; NZ_JADMSQ010000098.1; NZ_WQQY01000001.1; NZ_JAKNHU010000046.1; NZ_JAJCJS010000047.1; NZ_JAHYPM010000077.1; NZ_JAQDJF010000007.1; NZ_JAQDJE010000006.1; NZ_JAFBJH010000021.1; NZ_JAFBJF010000021.1	1	7
Phoceicola vulgatus	544	97.0-100	100	NZ_QSSN01000021.1; NZ_QSBO01000007.1; NZ_JADPEZ010000016.1; NZ_QRMN01000001.1; NZ_QROU01000027.1; NZ_JAKKXJ010000056.1; NZ_JAKKXN010000001.1; NZ_JAKKXS010000056.1; NZ_JAKKWW010000002.1; NZ_JAKKXI010000074.1; NZ_JAKKXB010000001.1; NZ_JANUTN010000007.1; NZ_JAHYRE010000075.1; NZ_QRYD01000047.1; NZ_QRYF01000060.1; NZ_JAHOIR010000010.1; NZ_JAHOIT010000010.1; NZ_JAHOIU010000008.1; NZ_JAHOIM010000010.1; NZ_JAHOIW010000010.1; NZ_JAHOIX010000010.1; NZ_JAHOIV010000010.1; NZ_JAHOIS010000010.1; NZ_JAHOIL010000009.1; NZ_JAHOIN010000010.1	2	4
Porphyromonas somerae	4	100	100	NZ_AP025559.1	1	100
Sutterella wadsworthensis	62	100	100	NZ_JAANXS010000022.1	1	0
