# Supplementary material for: Inter-phylum circulation of a beta-lactamase-encoding gene: a rare but observable event
Source: Antimicrob Agents Chemother. 2024 Mar 5;68(4):e01459-23. doi: 10.1128/aac.01459-23 (PMC10989005; doi:10.1128/aac.01459-23)
Supplement: Table S5 — Hits obtained analyzing the blaMUN-1 gene distribution using MGnify. [file aac.01459-23-s0008.pdf]

Supplementary Table 5: Hits obtained analyzing the blaMUN-1 gene distribution using MGnify.

Accession	Catalogue	Type	Taxonomy	K-mers in query	K-mers found in genome	% K-mers found
MGYG000003681	human-gut-v2-0-1	Isolate	Bacteroides stercoris	798	798	100
MGYG000303511	pig-gut-v1-0	MAG	Onthomorpha sp016296345	798	798	100
MGYG000000215	human-gut-v2-0-1	Isolate	Prevotella stercorea	798	775	97.12
MGYG000003252	human-gut-v2-0-1	MAG	Bacteroides sp900761785	798	775	97.12
