# Supplementary material for: Inter-phylum circulation of a beta-lactamase-encoding gene: a rare but observable event
Source: Antimicrob Agents Chemother. 2024 Mar 5;68(4):e01459-23. doi: 10.1128/aac.01459-23 (PMC10989005; doi:10.1128/aac.01459-23)
Supplement: Table S6 — Hits obtained analyzing the MUN-1 protein distribution using the GMGC catalog. [file aac.01459-23-s0009.pdf]

Supplementary Table 6: Hits obtained analyzing the MUN-1 protein distribution using the GMGC catalog.

Unigene	E-value	Complete	Habitat	Taxon (predicted)
GMGC10.047_051_980.UNKNOWN	4.76e-154	1	human gut,human nose,human oral,human skin,human vagina,mouse gut	Prevotellamassilia timonensis (species)
GMGC10.047_909_394.UNKNOWN	4.93e-143	1	human gut	Prevotellamassilia timonensis (species)
GMGC10.051_471_042.UNKNOWN	4.93e-143	1	human gut	Prevotellamassilia timonensis (species)
GMGC10.302_260_782.UNKNOWN	8.71e-140	1	human gut	Prevotellamassilia timonensis (species)
GMGC10.203_264_893.UNKNOWN	6.9e-137	1	human gut	Prevotellamassilia timonensis (species)
GMGC10.207_492_799.UNKNOWN	4.49e-120	1	human gut	Prevotellamassilia timonensis (species)
GMGC10.306_704_321.UNKNOWN	3.45e-112	1	human gut	Prevotellamassilia timonensis (species)
GMGC10.052_355_696.UNKNOWN	1.31e-103	1	cat gut,human gut	Prevotellamassilia timonensis (species)
GMGC10.190_096_469.UNKNOWN	5.16e-100	1	human gut,pig gut	Prevotellamassilia timonensis (species)
GMGC10.209_122_821.UNKNOWN	2.56e-99	1	human gut	Prevotellamassilia timonensis (species)
GMGC10.206_835_329.UNKNOWN	3.13e-97	1	human gut	Parabacteroides johnsonii DSM 18315 (species)
GMGC10.050_469_855.UNKNOWN	7.72e-96	1	human gut	Prevotellamassilia timonensis (species)
GMGC10.208_088_023.UNKNOWN	1.51e-91	1	human gut	Parabacteroides johnsonii DSM 18315 (species)
GMGC10.055_103_744.UNKNOWN	1.32e-87	1	human gut	Parasutterella excrementihominis YIT 11859 (species)
GMGC10.206_640_283.UNKNOWN	6.55e-87	1	human gut	Alistipes sp. CAG:831 (species)
GMGC10.001_916_490.UNKNOWN	1.23e-85	0	dog gut	Parabacteroides goldsteinii (species)
GMGC10.050_503_506.UNKNOWN	2.58e-83	1	human gut	Bacteroides sp. CAG:144 (species)
GMGC10.000_544_401.UNKNOWN	1.67e-82	1	dog gut,human gut	Parasutterella excrementihominis YIT 11859 (species)
GMGC10.147_033_135.UNKNOWN	1.85e-81	1	human gut,mouse gut	Parasutterella excrementihominis YIT 11859 (species)
GMGC10.056_912_393.UNKNOWN	2.41e-81	1	human gut,mouse gut	Parasutterella excrementihominis YIT 11859 (species)
GMGC10.210_339_896.UNKNOWN	5.37e-81	1	human gut,mouse gut	Parasutterella excrementihominis YIT 11859 (species)
GMGC10.054_684_518.UNKNOWN	9.16e-81	1	human gut	Bacteroides sp. CAG:144 (species)
GMGC10.055_669_095.UNKNOWN	1.2e-80	1	human gut,mouse gut	Parasutterella excrementihominis YIT 11859 (species)
GMGC10.306_210_464.UNKNOWN	1.01e-79	1	human gut	Parasutterella excrementihominis YIT 11859 (species)
GMGC10.310_770_158.UNKNOWN	9.5e-70	1	cat gut,human gut,human nose,human vagina	Bacteroides sp. CAG:20 (species)
GMGC10.306_122_048.UNKNOWN	5.21e-68	1	human gut	Bacteroides timonensis (species)
GMGC10.184_778_431.UNKNOWN	1.52e-67	1	human gut,human oral	Bacteroides timonensis (species)
GMGC10.309_096_849.UNKNOWN	1.28e-66	1	human gut,human oral,mouse gut	Bacteroides intestinalis (species)
GMGC10.207_343_478.UNKNOWN	1.68e-66	0	human gut,human oral,mouse gut	Bacteroides timonensis (species)
GMGC10.207_362_373.UNKNOWN	1.68e-66	0	human gut,human oral,mouse gut	Bacteroides timonensis (species)
GMGC10.055_127_397.UNKNOWN	2.86e-66	1	human gut	Bacteroides stercorirosoris (species)
GMGC10.032_188_571.UNKNOWN	1.09e-65	1	-	Bacteroides stercorirosoris (species)
GMGC10.177_802_870.UNKNOWN	1.62e-61	1	mouse gut	Bacteroidales (order)
GMGC10.298_506_683.UNKNOWN	2.12e-61	1	human gut	Odoribacter laneus CAG:561 (species)
GMGC10.207_032_025.UNKNOWN	2.77e-61	1	human gut	Parabacteroides timonensis (species)
GMGC10.297_552_894.UNKNOWN	4.73e-61	0	human gut	Bacteroidales (order)
GMGC10.207_524_736.UNKNOWN	8.06e-61	1	human gut	Parabacteroides timonensis (species)
GMGC10.057_236_757.UNKNOWN	2.35e-60	1	human gut	Parabacteroides timonensis (species)
GMGC10.050_713_307.UNKNOWN	1.52e-59	1	human gut	Bacteroides sp. Marseille-P3108 (species)
GMGC10.174_742_840.UNKNOWN	7.54e-59	1	human gut,mouse gut	Bacteroides (genus)
GMGC10.184_003_774.UNKNOWN	7.54e-59	1	human gut,mouse gut	Bacteroides congonensis (species)
GMGC10.306_142_568.UNKNOWN	1.68e-58	1	cat gut,dog gut,human gut,human nose,human oral,human skin,human vagina,mouse gut	Bacteroides vulgatus (species)
GMGC10.256_862_127.SP_0010	2.2e-58	1	human gut	Bacteria (superkingdom)
GMGC10.206_992_817.UNKNOWN	2.87e-58	1	human gut,mouse gut	Bacteroides congonensis (species)
GMGC10.288_000_481.UNKNOWN	1.09e-57	0	cat gut,dog gut,human gut,human nose,human oral,human skin,human vagina,mouse gut	Bacteroides dorei 5_1_36/D4 (species)
GMGC10.177_589_833.UNKNOWN	1.86e-57	1	mouse gut	Bacteroides (genus)
GMGC10.211_900_450.UNKNOWN	4.14e-57	1	human gut	Parabacteroides timonensis (species)
GMGC10.050_299_762.UNKNOWN	5.41e-57	1	human gut,mouse gut	Bacteroides acidifaciens JCM 10556 (species)
GMGC10.178_223_713.UNKNOWN	5.41e-57	1	human gut,mouse gut	Bacteroides acidifaciens JCM 10556 (species)
GMGC10.177_403_137.UNKNOWN	7.06e-57	1	mouse gut	Alistipes obesi (species)
